# Effects of *Cyperus rotundus* root exudates on *Ralstonia solanacearum* and *Bacillus velezensis* in relation to tobacco bacterial wilt occurrence

**DOI:** 10.1128/spectrum.00208-26

**Published:** 2026-07-23

**Authors:** Hongmei Wu, Jinyi Lu, Haofeng Wu, Shihong Liu, Lijuan Peng, Yuhan He

**Affiliations:** 1 College of Tobacco Science of Guizhou University, Guizhou Provincial Key Laboratory for Tobacco Quality Improvement and Efficiency Enhancement, Guiyang, China; Institute of Eco-environmental and Soil Sciences, Guangdong Academy of Sciences, Guangdong, China

**Keywords:** tobacco bacterial wilt, *Cyperus rotundus*, root exudates, rhizosphere microorganisms

## Abstract

Root exudates, functioning as chemical signaling molecules in the rhizosphere, regulate the structure and function of rhizosphere microbial communities, and play vital roles in plant disease resistance. This study characterized the rhizosphere microecological characteristics of tobacco and its accompanying weed *Cyperus rotundus* across different disease stages and explored the mechanisms by which *C. rotundus* root exudates affect *Ralstonia solanacearum* and its antagonistic strain *Bacillus velezensis*. The results showed that as soil temperature, humidity, and concurrent increases in *R. solanacearum* in the tobacco rhizosphere increased, the severity of tobacco bacterial wilt disease intensified. Additionally, the α-diversity of the rhizosphere soil microbial community exhibited a declining trend as the disease progressed. The rhizosphere of *C. rotundus* was more conducive to the stable enrichment of disease-suppressing bacterial groups such as Actinobacteria and Acidobacteria. In contrast, beneficial bacterial groups in the tobacco rhizosphere, including the phylum *Pseudomonadota* and the *Burkholderia*–*Caballeronia*–*Paraburkholderia* complex, declined continuously as the disease progressed. The active substances exerted significantly different effects on the two bacterial strains. At 50 μM, ferulic acid and phenylglyoxylic acid promoted the growth, biofilm formation, and motility of *R. solanacearum*. At 150 μM, phenylglyoxylic acid and 2-methoxycinnamaldehyde promoted the growth and motility of *B. velezensis*. Pot experiments confirmed that phenylglyoxylic acid and 2-methoxycinnamaldehyde at concentrations above 150 μM exerted certain control effects against bacterial wilt. In conclusion, the combined effects of soil environmental factors and root exudates alter the rhizosphere microbial community structure, creating a favorable microenvironment for *R. solanacearum* proliferation and thereby accelerating disease progression.

**IMPORTANCE:**

Interactions between plants and rhizosphere microbial communities are key factors governing plant resistance to pathogen stress. This study investigated the regulatory effects of *C. rotundus* root exudates on *R. solanacearum* and its antagonistic bacteria from the perspective of tripartite interactions among weeds, pathogens, and crops, thereby providing new insights into the microecological regulatory mechanisms of tobacco bacterial wilt. Furthermore, *C. rotundus* exhibited an enrichment effect on *R. solanacearum* at the early disease stage, while it selectively reshaped the rhizosphere microbiome via recruiting beneficial microbial communities during critical disease development stages. These findings deepen the understanding of the mechanisms by which associated weeds influence crop diseases and provide theoretical support for green prevention and control strategies against bacterial wilt.

## INTRODUCTION

In agricultural ecosystems, field weeds not only compete with crops for resources (1) but can also serve as reservoirs for pathogens, thereby increasing the survival ability of pathogenic bacteria in the absence of hosts (2). Pathogens can continuously infect susceptible hosts or colonize the rhizosphere of nonhost plants (3). For example, *Ralstonia solanacearum* can colonize the roots of various weeds, such as *Cyperus rotundus* (4), *Plantago asiatica* (5), and *Bidens pilosa* (6). Greater microbial community diversity facilitates the construction of a more complex microbial co-occurrence network, thereby enhancing the plant’s ability to resist pathogen invasion (7). When plants are infected by pathogens, their roots, leaves, or other tissues can regulate the chemical composition of their secretions, further influencing the community structure of plant-associated microorganisms (8), indicating that weeds play a significant role in the transmission dynamics of bacterial wilt. *C. rotundus* is one of the most common and invasive weeds in cultivated fields (9). Compared with C_3_ plants, C_4_ plants can utilize low concentrations of CO_2_ more efficiently under high-temperature environments. Therefore, under continuous high-temperature and high-humidity conditions, compared with tobacco and other weeds, *C. rotundus* grows more vigorously (10). Moreover, high-temperature (11) and high-humidity (12, 13) environments further enhance the activity of *R. solanacearum*, promoting its reproduction and increasing its transmission and pathogenic capabilities. *R. solanacearum* can be recruited by the root exudates of *C. rotundus* and utilize these exudates for survival and proliferation. The bacteria can colonize and overwinter in their tubers, serving as the primary source of infection for the disease in the following year, thus exacerbating the risk of tobacco bacterial wilt outbreaks (14, 15).

Root exudates, as crucial mediators connecting plants, environmental microorganisms, and environmental conditions (16), direct microorganisms to migrate toward the rhizosphere soil and root surfaces through chemotaxis, thereby driving interactions between plants and soil microorganisms (17). Plants can recruit beneficial microorganisms via root exudates, thereby increasing their stress resistance and disease resistance. For example, tobacco plants infected with *R. solanacearum* may secrete increased levels of chemotactic inducers to recruit resistant bacteria, thereby defending against pathogen invasion (18). Conversely, pathogens can also utilize the chemotactic properties of root exudates to colonize, infect, and reproduce within host plants (19). For instance, *R. solanacearum* can utilize L-glutamic acid from plant secretions to enhance its chemotaxis (motility) and promote biofilm formation, thereby strengthening its pathogenicity (20). Currently, research on the root exudates of *C. rotundus* is relatively limited and primarily focuses on the identification of phytochemical constituents in rhizome extracts, investigations into pharmacological activity, and clinical applications (21). Moreover, discussions on the mechanisms of action on soil rhizosphere bacteria remain scarce. In this study, rhizosphere soils from tobacco plants and their surrounding *C. rotundus* were collected at different disease severity levels. The collected soil samples were subjected to soil amplicon sequencing, qPCR quantitative analysis of bacterial wilt pathogens, and strain isolation and identification. Additionally, metabolomic profiling of *C. rotundus* root exudates was performed to investigate their effects on the growth, biofilm formation, and motility of *R. solanacearum* and its antagonistic bacteria. Based on integrated multi-omics analysis, this study aims to explore the intrinsic relationship between root exudates of *C. rotundus* and the spread process of *R. solanacearum* and to reveal the core mechanism by which these exudates regulate the communities of pathogenic and antagonistic bacteria.

## MATERIALS AND METHODS

### Soil sample collection

Rhizosphere soils of tobacco plants at different disease stages, as well as those of the weed *C. rotundus* growing around tobacco plants, were collected from Yangwu Town, Anshun City, Guizhou Province, China. The sampling site was characterized by flat terrain with uniform soil fertility, where tobacco had been continuously cultivated for many years and had a history of bacterial wilt disease occurrence. The site has an altitude of 1153 m, with geographical coordinates of 26°03′N latitude and 106°19′E longitude. The soil had a pH of 4.7, with an organic matter content of 44.36 g/kg, a total nitrogen content of 2.47 g/kg, a total phosphorus content of 1.88 g/kg, a total potassium content of 9.07 g/kg, an alkali-hydrolyzable nitrogen content of 251.92 mg/kg, an available phosphorus content of 63.6 mg/kg, and an available potassium content of 356.38 mg/kg. The tobacco variety planted in this field was “Yunyan 87.” Rhizosphere soil samples were collected during four stages of tobacco bacterial wilt disease development: (i) the first sampling (May 20, 2024), when tobacco bacterial wilt had not yet appeared; (ii) the second sampling (June 15, 2024), when the disease first occurred; (iii) the third sampling (July 10, 2024), during severe disease incidence; and (iv) the fourth sampling (July 20, 2024), during the late stage of the disease. The samples collected during each period included tobacco rhizosphere soil and the rhizosphere soil of *C. rotundus* growing around the tobacco plants. Details of the samples are shown in Table 1. Before sampling, the top 1 cm of soil was removed (22), and a shovel was used to dig soil blocks at a depth of 2–15 cm around the plants. The plants were then uprooted (23), retaining the rhizosphere soil particles. After the samples were brought to the laboratory, soil adhering to a thickness of 2 mm was collected using a brush, which constituted the rhizosphere soil (24).

**TABLE 1:** Sampling times and sample numbers^*a*^

Sampling period	Period number	Corresponding period sample	Sample number
Preonset period	A	Healthy tobacco rhizosphere soil	A1-1
		Rhizome soil of *C. rotundus*	A1-2
Initial onset phase	B	Slightly diseased tobacco rhizosphere soil	B1-1
		Rhizome soil of *C. rotundus*	B1-2
Peak incidence period	C	Slightly diseased tobacco rhizosphere soil	C1-1
		Rhizome soil of *C. rotundus*	C1-2
		Infected tobacco rhizosphere soil	c1-1
		Rhizome soil of *C. rotundus*	c1-2
Late stage of the disease	D	Infected tobacco rhizosphere soil	D1-1
		Rhizome soil of *C. rotundus*	D1-2

^*a*^ Replicate samples were designated by appending sequential hyphens and numbers (e.g., “-1,” “-2,” “-3”) to the base sample identifier, as in “A1-1-1,” “A1-1-2,” and “A1-1-3”.

### Investigation of tobacco bacterial wilt and statistical analysis of soil temperature and humidity

After tobacco transplantation, a soil temperature and humidity recorder (brand: Maxim, model DS1923-F5#, China) was used to collect temperature and humidity data at a soil depth of 15 cm in the tobacco field every 30 min. From 30 to 90 days after transplanting, the occurrence of tobacco bacterial wilt was observed every 7 days. Concurrent investigations of tobacco bacterial wilt severity were performed at each soil sampling time point, with individual plants used as statistical units across four independent surveys. Disease incidence and disease index were recorded and calculated accordingly (25).

### Isolation of soil bacteria and DNA extraction

The isolation and identification of *R. solanacearum* and its antagonistic bacteria were conducted according to the methods of Lang Zhong and Yanxiao Liu (26, 27), with appropriate modifications. The isolation of *R. solanacearum* also employed the tissue separation method, which involved selecting tobacco plants infected with bacterial wilt and showing bacterial ooze. Bacterial ooze was directly picked from the diseased tissue and placed in a PE tube containing sterile water for dilution. After treatment, streaking and purification were performed on TTC medium (26) (peptone, 10.0 g/L; beef extract, 3.0 g/L; sodium chloride, 5.0 g/L; agar powder, 20.0 g/L; TTC, 0.01 g/L). Pathogenicity of the isolated strains was determined using the root-wounding and root-drenching method. The purified *R. solanacearum* was diluted with sterile water, and the bacterial suspension concentration was adjusted to 3 × 10^8^ CFU/mL. A 10 mL sterile syringe was used to puncture 6–8 holes at the base of each tobacco seedling stem, followed by inoculation with 20 mL of the *R. solanacearum* suspension. A control group was inoculated with 20 mL of sterile water. For the identification of *R. solanacearum*, specific primer pairs were used: RALS-F/RALS-R (5′-GCTCAAGGCATTCGTGTGGC-3′/5′-GTTCATAGATCCAGGCCATC-3′), 759-F/760-R (5′-GTCGCCGTCAACTCACTTTCC-3′/5′-GTCGCCGTCAGCAATGCGGAATCG-3′), RfliC-F/RfliC-R (5′-CCTCAGCCTCAATACCAACATC-3′/5′-CATGTTCGACGTTTCAGAAGC-3′), Nmult21:1F/Nmult22:RR (5′-CGTTGATGAGGCGCGCAATTT-3′/5′-TCGCTTGACCCTATAACGAGTA-3′), and 27F/534R (5′-AGAGTTTGATCCTGGCTCAG-3′/5′-ATTACCGCGGCTGCTGG-3′) for multiplex PCR amplification. Additionally, this study utilized the universal bacterial primers 27F/1492R (5′-AGAGTTTGATCCTGGCTCAG-3′/5′-GGTTACCTTGTTACGACTT-3′) to amplify the *16S* rRNA gene of the isolated *R. solanacearum*. A maximum likelihood method was employed to construct a phylogenetic tree for verification.

The isolation of antagonistic bacteria was performed using the dilution plate coating method. A 10 g soil sample was weighed and placed in a flask containing 90 mL of sterile water, and the flask was shaken in a constant-temperature incubator at 30°C and 200 r/min for 20 min. The sample was serially diluted with sterile water, and 100 μL of each dilution was spread onto NA solid medium (beef extract, 5.0 g/L; peptone, 10.0 g/L; sodium chloride, 5.0 g/L; agar powder, 20.0 g/L). Antagonistic bacteria were screened using the plate confrontation method (28). The identified *R. solanacearum* strain was activated, and a single colony was picked and cultured in NA liquid medium (without agar powder) at 30°C with shaking for 12 h to prepare a bacterial suspension of 3 × 10^8^ CFU/mL. The *R. solanacearum* suspension was added to NA medium at 40°C at a 1% inoculation rate and mixed thoroughly to prepare bacterial-containing plates. Each candidate bacterial strain suspension (3 μL) was spotted at four symmetrical points on the *Ralstonia*-containing plates, which were then incubated at 30°C for 24 h. The presence of inhibition zones was observed, and their sizes were measured. The same plate confrontation method was used for secondary screening of antagonistic strains with an inhibition radius exceeding 10 mm in the initial screening. The diameter of inhibition zones was measured and calculated as the diameter of the transparent inhibition area minus the diameter of the bacterial colony on the antagonistic plate. Identification of antagonistic bacteria involved amplifying universal bacterial primer pairs 27F/1492R (5′-AGAGTTTGATCCTGGCTCAG-3′/5′-GGTTACCTTGTTACGACTT-3′) and *gyr*A-1F/*gyr*A-1R (5′-CAGTCAGGAAATGCGTACGTCCTT-3′/5′-CAAGGTAATGCTCCAGGCATTGCT-3′), followed by construction of a phylogenetic tree using the maximum likelihood method.

Total DNA from all rhizosphere soil samples was extracted following the instructions of the E.Z.N.A. Soil DNA Kit (Omega Bio-tek, Norcross, GA, USA). The extracted DNA was verified by 1% agarose gel electrophoresis and quantified using a NanoDrop spectrophotometer.

### Quantitative detection of *R. solanacearum* and Illumina MiSeq high-throughput sequencing

To determine the absolute abundance of *R. solanacearum* in the soil samples, the specific primers 759F (5′-GTCGCCGTCAACTCACTTTCC-3′)/760R (5′-GTCGCCGTCAGCAATGCGGAATCG-3′) were used to quantify the number of *R. solanacearum* (29). The total amplification system volume was 30 μL and contained 15 μL of qPCR mix (Vazyme, China), 2 μL of DNA, 0.5 μL each of upstream and downstream primers, and 12 μL of ultrapure water. A standard curve was constructed using samples containing *16S* rRNA gene fragments, and their copy numbers were automatically calculated using an online calculator. Real-time quantitative polymerase chain reaction (qPCR) was performed using serial gradient dilutions of DNA fragments with known copy numbers, thereby obtaining a standard curve equation showing the relationship between gene copy numbers and cycle threshold (Ct) values. After the same polymerase chain reaction (PCR) run, the copy numbers of the samples were calculated on the basis of the standard curve equation and their own Ct values. Moreover, to determine the composition of soil bacterial communities at different disease stages, the V3–V4 region of the bacterial 16S rRNA gene was amplified using the primers 341F (5′-CCTAYGGGRBGCASCAG-3′) and 806R (5′-GGACTACNNGGGTATCTAAT-3′) (30). The purified PCR products were quantitatively analyzed using a Qubit 3.0 fluorometer (Life Invitrogen). Amplified samples with different tag sequences were mixed according to the principle of equal amounts. The pooled DNA products were used to construct Illumina paired-end (PE) sequencing libraries following the Illumina genomic sequencing library preparation workflow. The amplicon libraries were sequenced in PE250 mode on the Illumina platform according to standard procedures (31), and the library construction and sequencing tasks were completed by Shanghai LingEn Biotechnology Co., Ltd.

### Collection and component identification of *C. rotundus* root exudates

All the *C. rotundus* plants were collected from the same tobacco field, and the sampled plants were growing around the tobacco. A hydroponic direct extraction method (32) was used to collect root exudates from *C. rotundus*. The roots of the *C. rotundus* plants were washed and placed in culture bottles containing 500 mL of Hoagland nutrient solution. After 72 h of root repair, the roots were disinfected by soaking in 1% sodium hypochlorite for 5 min and then rinsed several times with sterile water. The cleaned plants were transferred into 200 mL light-proof conical flasks wrapped in aluminum foil, and sterile water was added to completely submerge the roots. They were cultured under natural light at 28–30°C for 72 h, after which the culture solution was collected. The collected liquid was filtered through Xinhua No. 1 filter paper, freeze-dried, and stored at −80°C. Root exudates collected from every 12 *C. rotundus* plants were combined as one biological replicate, and a total of 5 biological replicates were prepared. The five freeze-dried samples were ground into powder and separately packed into sample bags for storage. Nontargeted metabolomic analysis of *C. rotundus* root exudates was performed using LC‒MS ultrahigh-performance liquid chromatography (33), and this work was carried out by Wuhan MetWare Biotechnology Co., Ltd..

### Effects of the active substances of *C. rotundus* root exudates on bacteria

This study used the screening and identification results of *R. solanacearum* and its antagonistic strains to select strains with stable and optimal antagonistic effects against the pathogen, and explored the effects of active substances in *C. rotundus* root exudates on *R. solanacearum* and its antagonists based on their similar morphological and cultural characteristics. Studies on the effects of active substances in *C. rotundus* root exudates on bacterial growth, biofilm formation, and motility were conducted by referring to the experimental methods of Shili Li (34), Huicong Shi (35), and others, with appropriate adjustments. Bacterial growth (OD value) was determined following the turbidimetric method to quantitatively reflect the effect of active substances on strain growth. The effect on biofilm formation by the strain was measured following the crystal violet method. To investigate the effects of substances on strain motility, culture media with an agar concentration of 0.32%–0.35% were used, with emphasis on observing swarming motility to reflect the regulatory role of substances on bacterial motility. Each tested active substance was dissolved in absolute ethanol to prepare a 10 mM stock solution, which was sterilized by filtration through a 0.22 μm membrane filter and then stored at 4°C away from light for subsequent experiments.

In this study, both a semisynthetic medium (CPG nutrient medium [35]: glucose, 5 g/L; peptone, 10 g/L; acid hydrolyzed casein, 1 g/L; agar powder, 20.0 g/L, with liquid CPG not containing agar powder) and fully synthetic medium (M63 basic medium [34]: KH_2_PO_4_, 13.609 g/L; MgSO_4_·7H_2_O, 0.12 g/L; KOH, 4.2 g/L; FeSO_4_·7H_2_O, 0.000592 g/L; (NH_4_)_2_SO_4_, 1.98 g/L; glucose, 3.96 g/L) were used to determine the effects of each active substance on bacterial biomass. Different volumes of the active substance stock solutions were added to 30 mL of liquid CPG medium to prepare media containing final concentrations of 50 μM, 100 μM, 150 μM, and 200 μM. Equal volumes of absolute ethanol and sterile water were used as the solvent control and blank control, respectively. A 30 μL aliquot of bacterial culture with an OD_600_ of 1.0 was inoculated into each medium and incubated at 30°C with shaking at 200 r/min for 12 h and 24 h. The absorbance of the samples was measured at 600 nm, and the results were used to determine bacterial biomass. The experimental steps for M63 medium treatment were the same as those for CPG medium.

Five milliliters of each concentration of the drug-containing liquid CPG medium was dispensed into sterile 10 mL centrifuge tubes and inoculated with 5 μL of bacterial culture with an OD_600_ of 1.0. After thorough mixing, 200 μL of each treated culture was transferred into a sterile 96-well culture plate. The culture plate was placed in a 30°C constant temperature incubator for static cultivation for 12 h and 24 h. After cultivation, the culture plates were washed multiple times with sterile water to remove the cultures and allowed to dry at room temperature. Subsequently, 210 μL of 0.5% crystal violet solution was added to each well for staining. The samples were incubated at 25°C for 30 min and then washed several times with sterile water to remove the solution, after which they were allowed to air-dry at room temperature. After drying, 220 μL of 95% ethanol was added to each well, and the samples were sealed and incubated at 25°C for 10 min to dissolve the crystal violet on the biofilm. After complete dissolution, the absorbance of each well was measured at 590 nm using a microplate reader as an indicator of biofilm formation, and the amount of biofilm formed was determined after 12 h and 24 h of cultivation.

The effects of active substances on bacterial motility were determined using semisolid culture medium. The bacterial strains were cultured in the aforementioned drug-containing media at different concentrations and incubated at 30°C with shaking at 200 r/min. Subsequently, 1.5 μL of bacterial suspension was aspirated and dropped onto CPG semisolid agar (glucose, 0.508 g; hydrolyzed casein, 0.1 g; peptone, 1 g; agar powder, 0.325 g; purified water to 100 mL) plates. The plates were then placed in a 30°C incubator for 24 h. After incubation, colony spreading was observed, and the motility diameter was measured. The motility range was calculated by subtracting the diameter of the initial inoculation spot from the total diameter of the bacterial spread, and all diameters were measured in millimeters (mm).

Based on the experimental results regarding the effects of root exudate active substances on bacterial growth, biofilm formation, and motility, active substances that significantly inhibit the growth of *R. solanacearum* (AS-1) were selected for pot control experiments. The antagonistic bacterial suspension (JXF-16) treatment was used as the biological control, and the commonly used 45% kasugamycin–oxine-copper suspension concentrate (SC) in actual production was used as the chemical control. Through the dual settings of biological and chemical controls, the control effect of active substances on tobacco bacterial wilt was visually evaluated. The suspensions of AS-1 and JXF-16 were prepared with sterile water at concentrations of 1 × 10^7^ CFU/mL and 1 × 10^8^ CFU/mL, respectively. When the tobacco seedlings reached the five-leaf-one-heart stage, 20 mL of *R. solanacearum* suspension at a concentration of 1 × 10^7^ CFU/mL was inoculated using the root-wounding and root-drenching method. The treatments were as follows:

1. Root exudate active substance solution: After disease occurrence, on the basis of the concentration determined by the screening experiment, 20 mL of the solution was applied per plant via root drenching, with only one application.
2. Chemical pesticides: After disease onset, a 1,000-fold dilution of 45% kasugamycin–oxine-copper suspension concentrate (SC) was applied immediately. Each plant was irrigated with 20 mL of the solution, and the treatment was applied only once.
3. JXF-16 bacterial solution: Each tobacco seedling was root-irrigated with 20 mL of JXF-16 bacterial solution at a concentration of 1 × 10^8^ CFU/mL, treated once at the time of transplanting, and applied once again after the disease occurred, for a total of two treatments.
4. CK: Twenty milliliters of sterile water was applied to each tobacco seedling, which served as a control during each treatment application.

After the plants were inoculated with the pathogen *R. solanacearum*, the growth and disease incidence of the tobacco plants were observed every 12 h. Control measures were initiated immediately upon disease occurrence. Disease assessments were conducted every 3 days for a total of three investigations, and the incidence rate, disease index, and relative control efficacy were calculated (36).

### Data processing and statistical analysis

Analysis of α- and β-diversity and microbial community composition was performed using the bioinformatics cloud platform provided by LingEn Biotechnology Co., Ltd. (Shanghai, China). Shannon, Chao1, Pielou’s J, and other α-diversity indices were calculated using QIIME software. β-diversity was assessed using the Bray-Curtis distance matrix, and PERMANOVA was employed to test the differences in bacterial community structures among different rhizosphere soil samples. The *t*-test was used to compare the differences in the absolute abundance of *R. solanacearum* between samples. The effects of different active substances on the growth, biofilm formation, and motility of *R. solanacearum* and *B. velezensis* were evaluated using one-way ANOVA, with Tukey’s HSD method applied for multiple comparisons, and the significance level was set at *P* < 0.05.

## RESULTS

### Isolation and identification of *R. solanacearum* and *B. velezensis*

**Fig 1:**
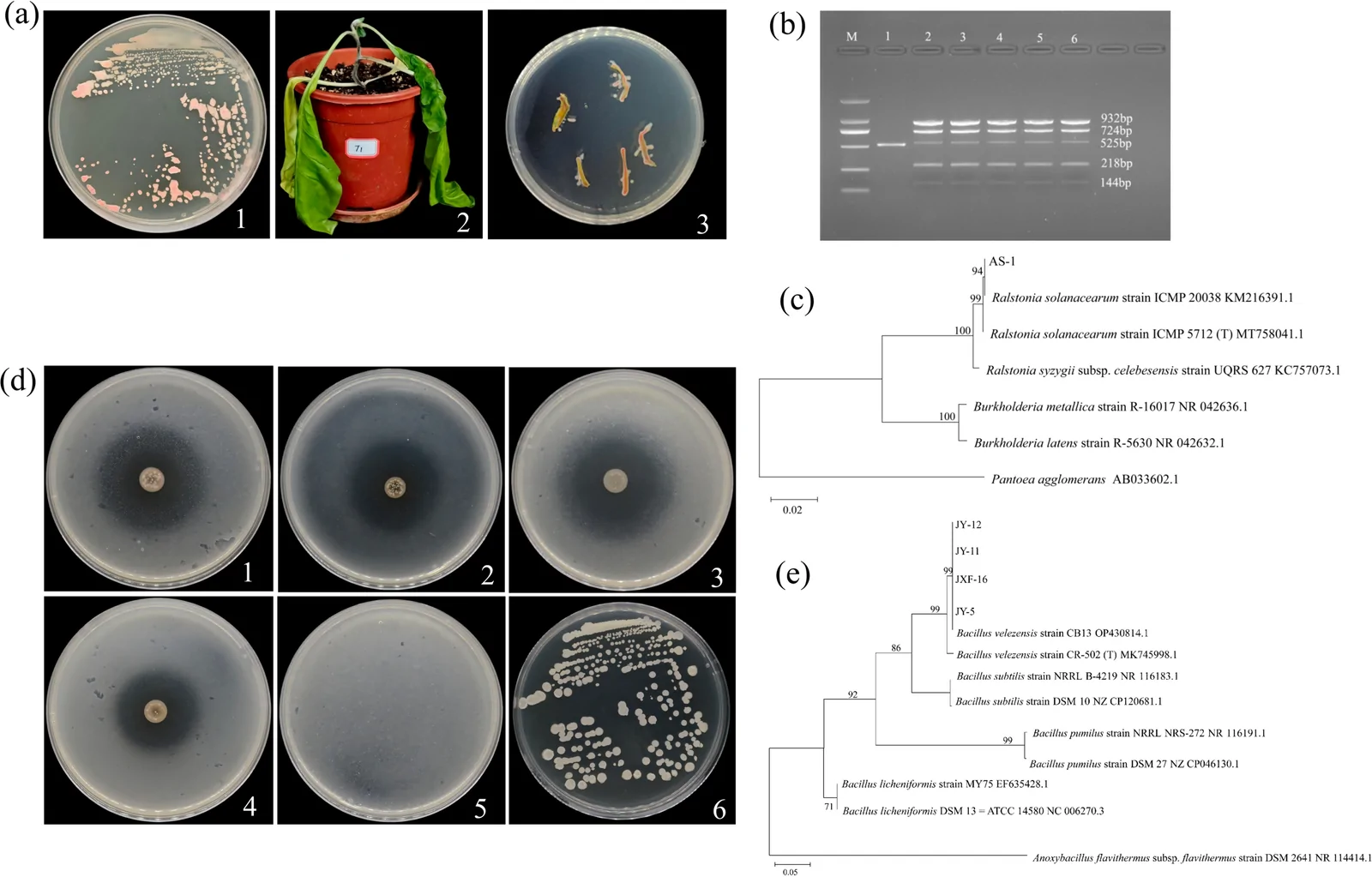
Isolation and identification of strains. (**a**) Experimental results of inoculating tobacco seedlings with the *R. solanacearum* strain AS-1. (1) Morphology of strain AS-1 grown on 2,3,5-trichlorotriphenyl-tetrazolium chloride (TTC) medium. (2) Tobacco seedlings exhibiting wilting symptoms after AS-1 infection. (3) Tissue isolated from susceptible tobacco seedlings was inoculated onto TTC medium, yielding typical *R. solanacearum* colony morphology. (**b**) Results of multiplex PCR identification using *Ralstonia solanacearum*-specific primers. In the figure, lane M represents a 2,000 bp DNA ladder, lane 1 is the *Escherichia coli* negative control, lane 2 is the *R. solanacearum* GMI1000 positive control, and lanes 3–6 are the isolated *R. solanacearum* strains to be tested. (**c**) Phylogenetic tree of strain AS-1 constructed based on the *16S* rRNA gene. (**d**) Screening of antagonistic bacteria. (1–5) Images of inhibition zones formed after co-culturing antagonistic strains JXF-16, JY-12, JY-5, and JY-11 with strain AS-1. (6) Colony morphology of strain JXF-16 on LB medium. (**e**) Phylogenetic trees of antagonistic strains JXF-16, JY-12, JY-5, and JY-11 based on *16S* rRNA and *gyr*A genes.

**Fig 2:**
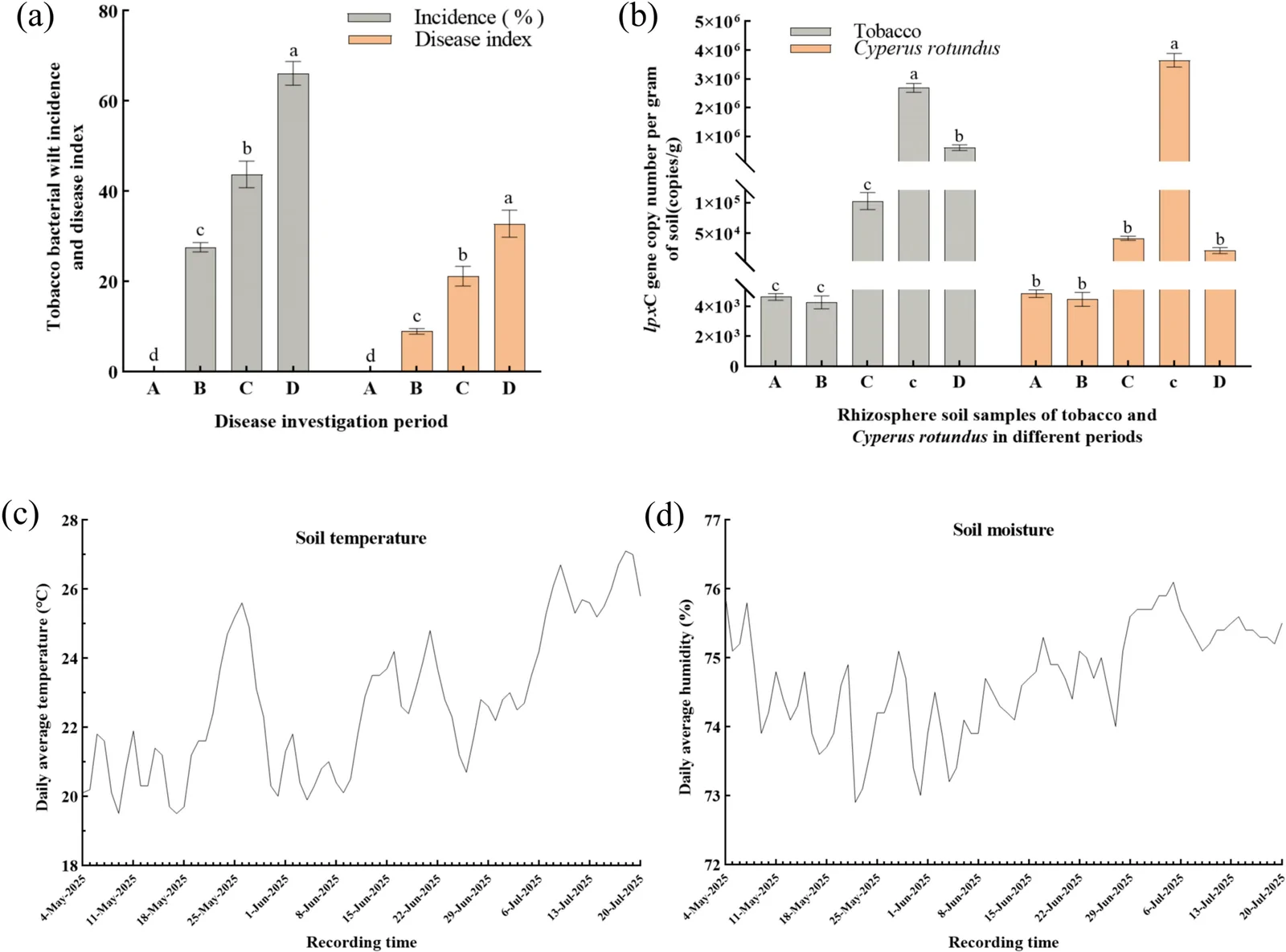
Relationships among the incidence of tobacco diseases, soil temperature, soil moisture, and copy number of the *lpx*C gene of *R. solanacearum.* (**a**) Field investigation results of tobacco bacterial wilt; letters on the x-axis indicate different sampling periods. (**b**) Copy numbers of bacterial wilt pathogen in the rhizosphere soil of tobacco and *C. rotundus* at different disease progression stages; different letters on the x-axis represent samples collected during the respective periods. (**c**) Daily average soil temperature at a depth of 15 cm from May to July. (**d**) Daily average soil moisture at a depth of 15 cm from May to July. The sampling codes A, B, C, c, and D on the x-axis of panels **a** and **b** correspond to samples from the pre-onset stage, initial disease stage, lightly infected samples at the peak disease stage, severely infected samples at the peak disease stage, and the late disease stage, respectively.

**Fig 3:**
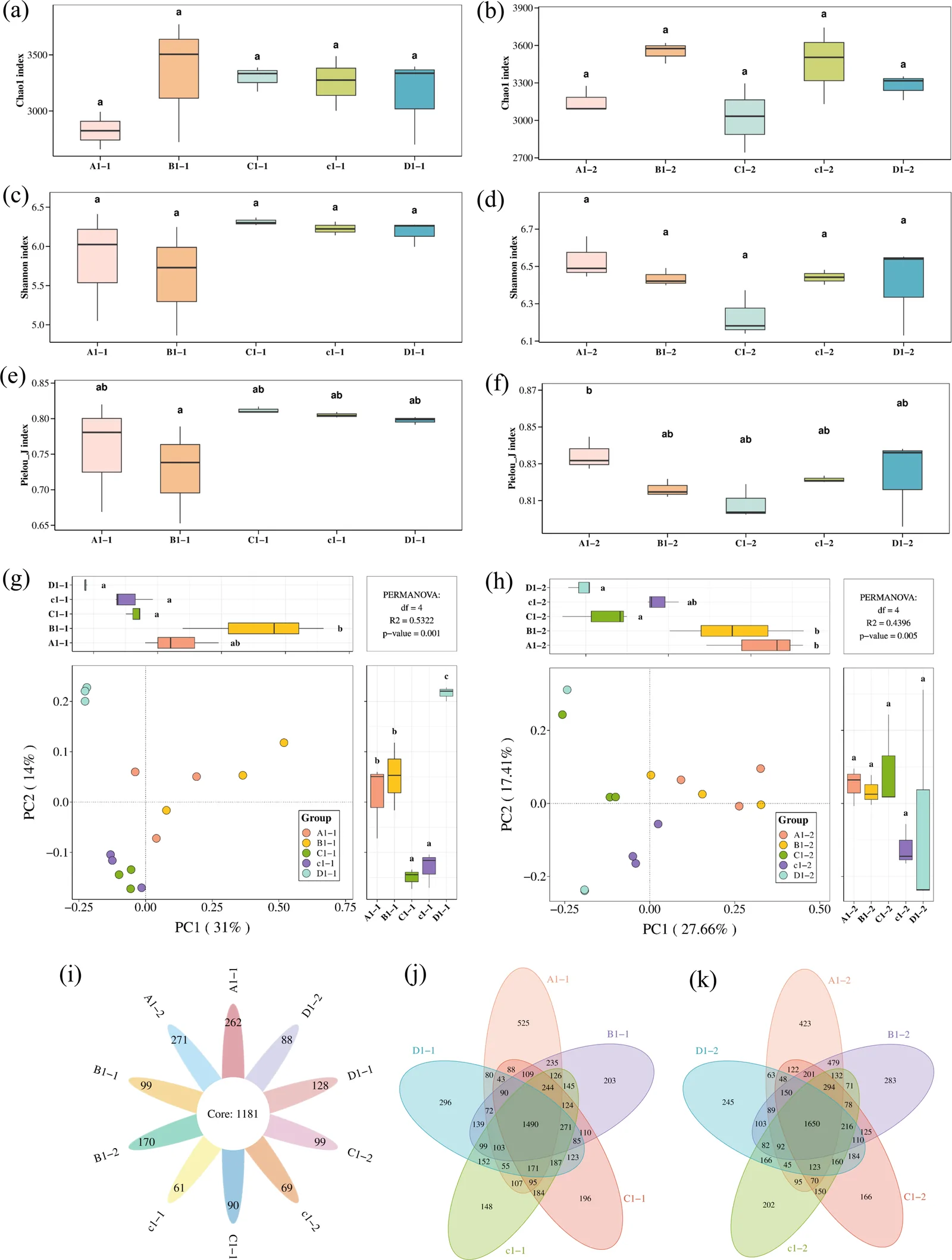
Analysis of microbial diversity and composition in the rhizosphere of tobacco and *C. rotundus.* The α-diversity of rhizosphere microbial communities was evaluated using the Chao1 index, Shannon index, and Pielou’s J evenness indices: boxplots of Chao1 for rhizosphere microorganisms of (**a**) tobacco and (**b**) *C. rotundus*; boxplots of Shannon for rhizosphere soil microorganisms of (**c**) tobacco and (**d**) *C. rotundus*; and boxplots of Pielou's J for rhizosphere soil microorganisms of (**e**) tobacco and (**f**) *C. rotundus*. β-diversity analysis of rhizosphere microbial communities in (**g**) tobacco and (**h**) *C. rotundus*. (**i**) OTU distribution of tobacco and *C. rotundus* samples during different periods. (**j**) OTU distribution of tobacco samples at different periods. (**k**) OTU distribution of *C. rotundus* samples at different time points. Sample codes in panels **a** to **k** are defined as follows: letters A, B, C, c, and D represent samples from the pre-disease stage, early disease stage, peak disease stage (C for mildly infected, c for severely infected), and late disease stage, respectively. The suffix “1-1” following each letter denotes tobacco rhizosphere soil samples, while “1-2” stands for *C. rotundus* rhizosphere soil samples.

After isolation and purification, raised, smooth colonies with pink centers and milky white margins, which were relatively broad, appeared on the TTC agar plates, consistent with the typical colony morphology of *R. solanacearum* (Fig. 1a). Following the application of *R. solanacearum* solution via the root-wounding and root-drenching method, the tobacco plants exhibited symptoms of black spots on the lower stems and wilting and necrosis of the lower leaves. The *R. solanacearum* strain was reisolated from the infected samples, and the results conformed to Koch’s postulates (Fig. 1a, 2 and 3). After performing multiplex PCR amplification on the genomic DNA of *R. solanacearum* using the specific primer pairs RALSF/RALSR, 759F/760R, RfliC-F/RfliC-R, Nmult21:1F/Nmult22:RR, and 27F/534R, specific bands with expected sizes (932 bp, 281 bp, 724 bp, 144 bp, and 525 bp, respectively) were obtained (Fig. 1b). Simultaneously, the *16S* rRNA gene sequence detection results were compared with the genomic sequences in the NCBI database, and a phylogenetic tree was constructed. The results showed that strain AS-1 shared 99% bootstrap support with the *R. solanacearum* type strain ICMP5712 and clustered together with *R. solanacearum* strain TCMP20038 with 94% bootstrap support. Combined with morphological characteristics and pathogenicity assays, the isolated strain was identified as the causal agent of tobacco bacterial wilt (*R. solanacearum*) (Fig. 1c).

Following plate confrontation experiments with strain AS-1, 20 bacterial strains exhibiting favorable antagonistic effects against *R. solanacearum* were preliminarily screened. After rescreening, the inhibition diameters of strains JY-5, JY-11, JY-12, and JXF-16 reached 38.08 mm, 26.68 mm, 30.07 mm, and 44.25 mm, respectively (Fig. 1d). Taking strain JXF-16 as an example, its colonies on LB plates appeared dry, flat, white, circular, and had irregular edges (Fig. 1d). Phylogenetic trees constructed based on the *16S* rRNA and *gyr*A gene sequences showed that strains JY-5, JY-11, JY-12, and JXF-16 shared 99% bootstrap support with the type strain *B. velezensis* CR-502 and also clustered with *B. velezensis* CB13 with similarly high bootstrap support of 99%. Accordingly, strains JY-5, JY-11, JY-12, and JXF-16 were identified as *B. velezensis* (Fig. 1e).

### The occurrence of tobacco bacterial wilt is significantly positively correlated with *R. solanacearum* abundance and soil temperature and humidity

The occurrence of bacterial wilt was significantly positively correlated (*P* < 0.05) with the population of *R. solanacearum* in the rhizosphere soil and with changes in soil temperature and humidity. As the rhizospheric abundance of *R. solanacearum*, together with soil temperature and humidity, rose, the disease index of tobacco bacterial wilt increased markedly. Field survey results revealed that no plants exhibiting symptoms of bacterial wilt were observed in the field on May 20 (period A). By June 15 (period B), sporadic disease plants began to appear, and the soil *R. solanacearum* copy number did not significantly differ from that in period A (Fig. 2a and b). From May 22 to 28, the soil temperature increased significantly, with the highest daily average temperature reaching 28.1°C. During the week before disease onset, the soil temperature continued to increase, while the soil humidity remained above 73% (Fig. 2c and d). These factors collectively formed the environmental triggers for the initial disease outbreak.

By July 10 (period C), disease severity had significantly intensified. During the preceding week, the soil temperature continued to increase, with the daily maximum average temperature reaching 29.1°C. The number of *R. solanacearum* in the soil was approximately 1 × 10³ greater than that in periods A and B. The high-temperature and high-humidity environment significantly promoted pathogen proliferation. By July 20, the disease had spread further, as the soil remained under sustained high temperature and humidity, further accelerating the proliferation of *R. solanacearum* and disease transmission. The number of *R. solanacearum* in the rhizosphere of *C. rotundus* was greater than that in the tobacco rhizosphere during periods A and B, and during phase C, the abundance of *R. solanacearum* in the rhizosphere of *C. rotundus* surrounding severely infected tobacco plants (c) was also higher than that in the tobacco rhizosphere. In periods C and D, the *R. solanacearum* count in the tobacco rhizosphere exceeded that in the *C. rotundus* rhizosphere. During period C, the bacterial counts of both rhizospheres peaked. Overall, the increase in the abundance of *R. solanacearum* aligned with the severity of tobacco disease, indicating that pathogen proliferation drove disease progression.

### Diversity and compositional characteristics of bacterial communities in the rhizosphere soils of tobacco and *C. rotundus*

After basic quality control filtering of the high-throughput raw sequences, a total of 1,075,253 valid *16S* rRNA sequences were obtained from all the tested rhizosphere soil samples. The α-diversity of the bacterial communities was evaluated. The results showed that there were no significant differences in Chao1, Shannon, and Pielous’s J indices of rhizosphere soil bacteria between tobacco plants and *C. rotundus* at different stages of tobacco bacterial wilt disease (*P* > 0.05), indicating that the α-diversity of rhizosphere soil bacteria was not affected by the disease. However, the overall richness showed a declining trend as the disease progressed. During the peak disease period (C), the Chao1 value of rhizosphere soil from *C. rotundus* surrounding severely diseased tobacco plants (c1-2) was 15.28% higher than that from *C. rotundus* near mildly diseased tobacco plants (C1-2), and both were higher than those of tobacco rhizosphere samples from the same period (Fig. 3a and b). There were no significant differences in Shannon indices among rhizosphere soils of tobacco and *C. rotundus* across different periods (*P* > 0.05). However, during the initial disease stage (B), the Shannon index of *C. rotundus* rhizosphere soil was 14.66% higher than that of tobacco rhizosphere soil from the same period (Fig. 3c and d). Analysis of the Pielou’s J index revealed that the evenness of tobacco rhizosphere communities was slightly higher during stage B than during other periods, while the evenness of *C. rotundus* rhizosphere communities remained relatively stable across all stages (Fig. 3e and f).

To better understand how bacterial wilt affects bacterial communities, the β-diversity and community composition differences in the rhizosphere soil bacterial communities associated with tobacco and *C. rotundus* were compared across different stages. Principal coordinate analysis (PCoA) based on Bray-Curtis distance showed significant differences in the rhizosphere soil bacterial communities of tobacco at different stages (adonis test, *P* = 0.001, *R*² = 0.5322) (Fig. 3g and h). Samples from the initial disease stage (B) were distinctly separated from those of the peak disease stage (C) and late disease stage (D) along the PC1 axis, indicating significant differences in the rhizosphere soil microbial community structure between this stage and the other two stages. Along the PC2 axis, significant differences were observed between stages A and B and stages C and D. The rhizosphere soil bacterial community structure of *C. rotundus* explained a total of 45.07% of the variation across the two principal coordinates, with samples from stages A and B also showing clear separation from those of stages C and D along the PC1 axis. Venn analysis revealed that 1,181 common OTUs were identified across all rhizosphere soils. Among these, the rhizosphere soils of tobacco and *C. rotundus* in the asymptomatic stage (A) had the highest number of unique OTUs, indicating a higher level of microbial diversity during this period (Fig. 3i). The rhizosphere soils of tobacco across all stages shared 1,490 OTUs (Fig. 3j), while those of *C. rotundus* shared 1,650 OTUs (Fig. 3k). *C. rotundus* samples from stages A and B shared a greater number of OTUs, suggesting a certain similarity in community structure between these two stages.

**Fig 4:**
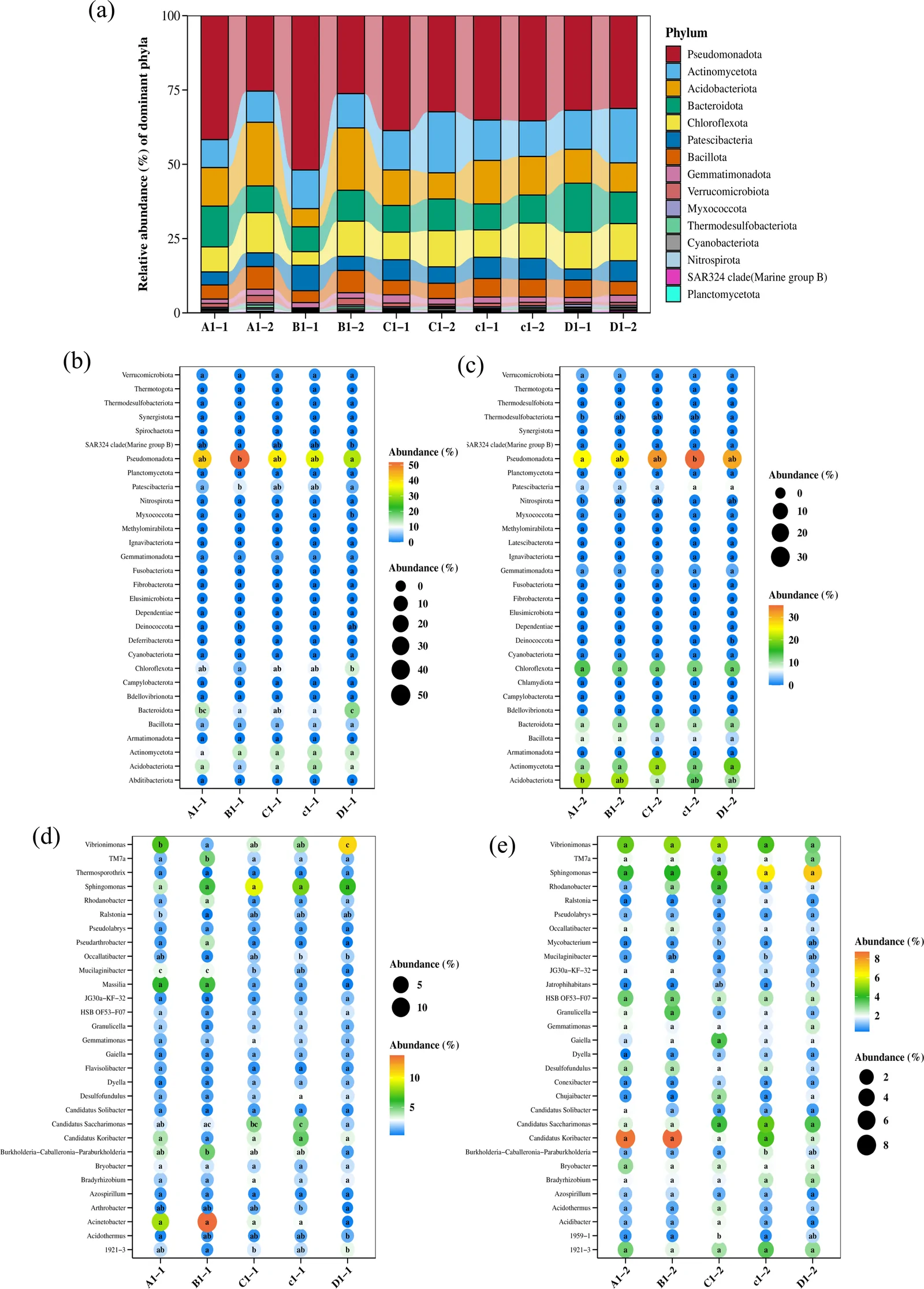
Analysis of differences in rhizosphere microbial community composition between tobacco and *C. rotundus.* (**a**) Stacked impact diagram of the species composition of rhizosphere microorganisms at the phylum level. Bubble charts showing differences in species composition at the phylum level for (**b**) tobacco and (**c**) *C. rotundus* rhizosphere microorganisms; bubble charts showing differences in species composition at the genus level for (**d**) tobacco and (**e**) *C. rotundus* rhizosphere microorganisms. The rows represent species, and the columns represent groups. The size and color of the circles correspond to the average relative abundance of the species in the corresponding group, and the lowercase letters within the circles represent the results of significance tests for differences in species abundance between groups. Sample codes in panels **a** to **e** are defined as follows: letters A, B, C, c, and D represent samples from the pre-disease stage, early disease stage, peak disease stage (C for mildly infected, c for severely infected), and late disease stage, respectively. The suffix “1-1” following each letter denotes tobacco rhizosphere soil samples, while “1-2” stands for *C. rotundus* rhizosphere soil samples.

Through comparison and analysis with the RDP database sequences, a total of 43 bacterial phyla were annotated across all samples. Among them, *Pseudomonadota*, *Actinomycetota*, *Acidobacteriota*, *Bacteroidota*, *Chloroflexota*, *Patescibacteria*, and *Bacillota* were the dominant phyla, accounting for 93.97% of the total sequences (Fig. 4a). *Pseudomonadota* was the absolute dominant phylum, with a relative abundance of 31.72%–51.74% in the tobacco rhizosphere, which decreased as the disease severity increased. In contrast, its relative abundance in the *C. rotundus* rhizosphere was 25.22%–35.20%, showing an upward trend with disease progression, but its overall abundance was lower than that in tobacco. The relative abundance of *Acidobacteriota* in the *C. rotundus* rhizosphere (8.76%–21.28%) was significantly higher than that in tobacco (6.08%–14.59%), with particularly notable differences during periods A and B. *Actinomycetota* remained relatively stable in the tobacco rhizosphere across all periods (approximately 13.0%), but increased significantly in the *C. rotundus* rhizosphere during periods C and D, reaching 20.47% and 18.19%, respectively. *Bacteroidota* in tobacco period D rose significantly to 16.42%, markedly higher than in *C. rotundus* during the same period, while *C. rotundus* maintained levels of 8.90%–10.67% across all periods. *Chloroflexota* was consistently higher in the *C. rotundus* rhizosphere (11.80%–13.48%) than in tobacco and showed a gradual upward trend with disease progression. Additionally, phyla such as *Bacillota*, *Myxococcota*, *Nitrospirota*, and *Verrucomicrobiota* exhibited higher overall abundances in the *C. rotundus* rhizosphere. *Patescibacteria* was significantly enriched in tobacco period B, while in *C. rotundus*, it gradually increased with disease progression (Fig. 4b and c).

Annotation was conducted at the genus level, resulting in a total of 1,165 genera. Analysis of the top 30 dominant genera (relative abundance >1%) revealed that *R. solanacearum*, the pathogen causing tobacco bacterial wilt, exhibited higher relative abundance in the tobacco rhizosphere compared to the *C. rotundus* rhizosphere across different periods. However, previously studied and reported antagonistic bacterial groups against *R. solanacearum* showed varying trends in this study: *Sphingomonas* significantly increased in abundance (9.39%, 7.52%) in tobacco samples with different disease severity during period C (samples C and c), while *C. rotundus* reached peak abundance (6.58%, 7.18%) during period C (sample c) and period D, representing core antagonistic groups during the peak disease phase. *Burkholderia*–*Caballeronia*–*Paraburkholderia* showed the highest abundance (4.96%) in tobacco during period B, followed by a significant decline as the disease progressed, whereas in the *C. rotundus* rhizosphere, its abundance increased during period C (sample c) to 2.25%. *Mucilaginibacter* exhibited higher abundance in tobacco during periods A and B but decreased markedly post-disease onset, whereas in the *C. rotundus* rhizosphere, its abundance showed a gradual upward trend, peaking during period C (sample c). Beneficial genera such as *Gemmatimonas*, *Bradyrhizobium*, *Arthrobacter*, *Acidothermus*, and *Gaiella* were upregulated to varying degrees during the peak disease phase. Additionally, *Acinetobacter* was enriched in the tobacco rhizosphere, peaking during period B (13.78%), and *Vibrionimonas* significantly increased during period D (10.93%), whereas these two groups remained stable across all periods in *C. rotundus. Candidatus Koribacter* and *Granulicella* were notably enriched in *C. rotundus* during periods A and B (Fig. 4d and e).

### Main components of *C. rotundus* root exudates and their effects on *R. solanacearum* and *B. velezensis*

**Fig 5:**
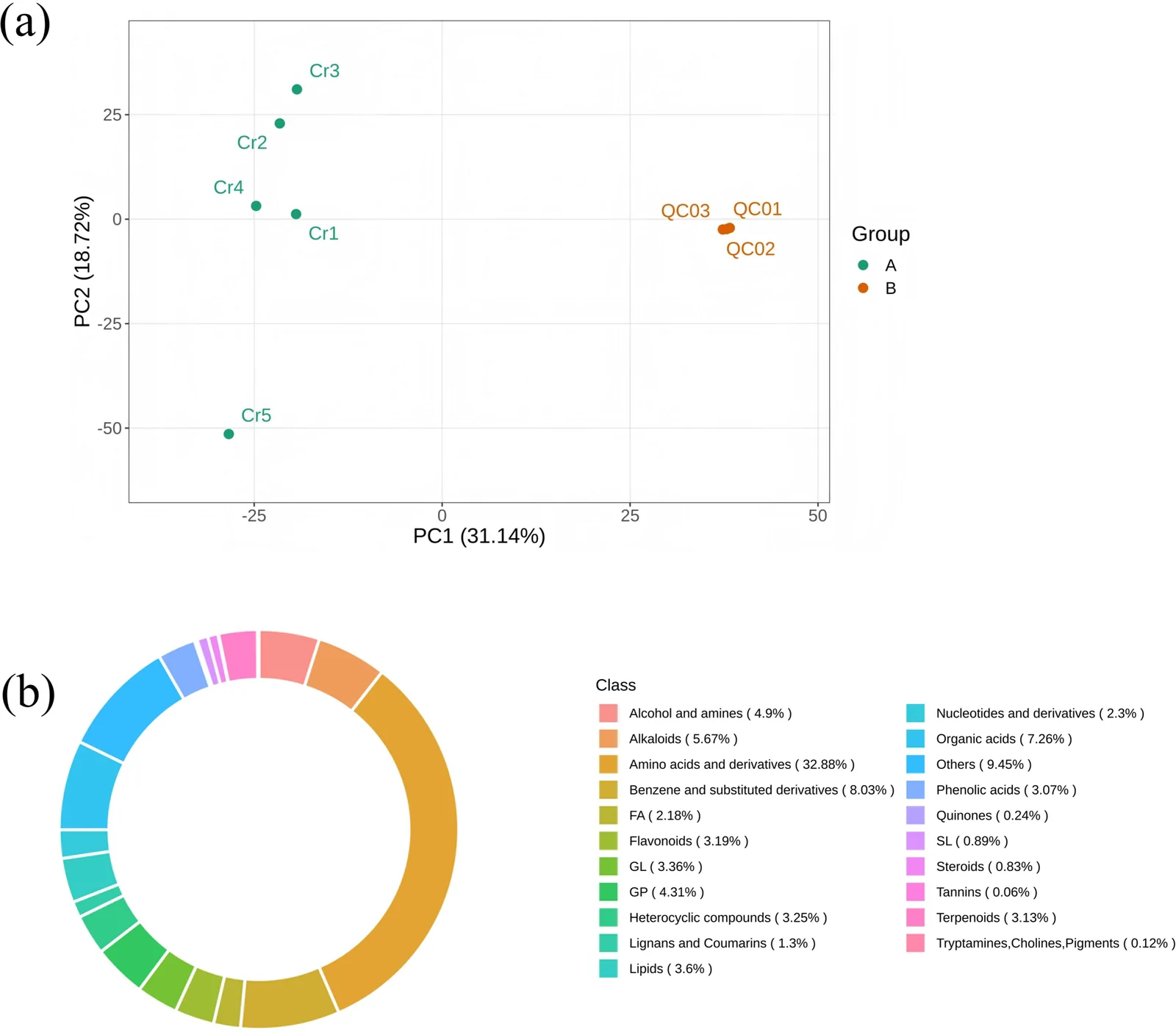
Composition analysis of *C. rotundus* root exudates. (**a**) PCA score plot of experimental samples and quality control samples. Each dot represents one sample; samples belonging to the same group are shown in the same color. Group A indicates experimental samples, and Group B indicates quality control samples prepared by pooling extracts from all experimental samples. (**b**) Pie chart showing the metabolite classification composition.

PCA was performed on the samples of *C. rotundus* root exudates. The reproducibility of the five root exudate samples was good (Fig. 5a), with small intragroup differences and no deviating samples. Analysis of the LC‒MS detection results revealed a total of 21 classes of compounds, encompassing 3,176 metabolites (Fig. 5b). Amino acids and their derivatives accounted for the most significant proportion (22.27%), followed by organic acids (13.43%). The composition of *C. rotundus* root exudates was analyzed, with screening criteria based on relative content, safety, functional potential, accessibility, and application cost. p-Toluenesulfonic acid, ferulic acid, phenylglyoxylic acid, and 2-methoxycinnamaldehyde were selected for subsequent studies to investigate their effects on *R. solanacearum* (AS-1) and *B. velezensis* (JXF-16).

#### Effects of four active compounds in *C. rotundus* root exudates on the growth of AS-1 and JXF-16

**Fig 6:**
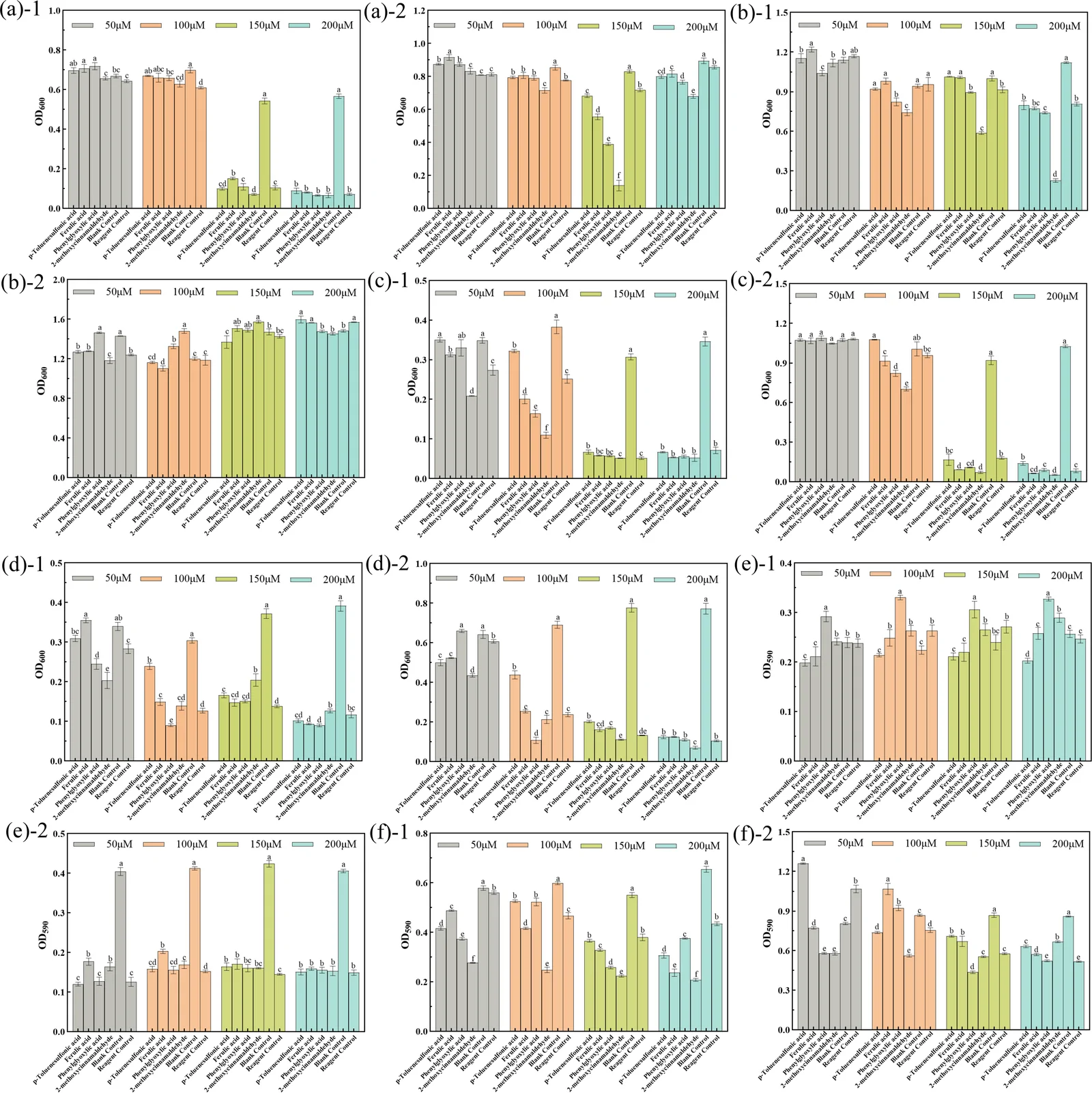
Effects of four active compounds from *C. rotundus* root exudates on bacterial growth and biofilm formation. (a)-1, growth of *R. solanacearum* cultured for 12 h in CPG medium supplemented with active compounds; (**a**)-2, growth of *R. solanacearum* cultured for 24 h in CPG medium supplemented with active compounds; (**b**)-1, growth of *B. velezensis* cultured for 12 h in CPG medium supplemented with active compounds; (**b**)-2, growth of *B. velezensis* cultured for 24 h in CPG medium supplemented with active compounds; (**c**)-1, growth of *R. solanacearum* cultured for 12 h in M63 medium supplemented with active compounds; (**c**)-2, growth of *R. solanacearum* cultured for 24 h in M63 medium supplemented with active compounds; (**d**)-1, growth of *B. velezensis* cultured for 12 h in M63 medium supplemented with active compounds; (**d**)-2, growth of *B. velezensis* cultured for 24 h in M63 medium supplemented with active compounds; (**e**)-1, biofilm formation of *R. solanacearum* cultured for 12 h in CPG medium supplemented with active compounds; (**e**)-2, biofilm formation of *R. solanacearum* cultured for 24 h in CPG medium supplemented with active compounds; (**f**)-1, biofilm formation of *B. velezensis* cultured for 12 h in CPG medium supplemented with active compounds; (**f**)-2, biofilm formation of *B. velezensis* cultured for 24 h in CPG medium supplemented with active compounds. Lowercase letters above each bar indicate significant differences among treatments with different active compounds.

Under nutrient medium conditions, at a concentration of 50 μM, the four active substances either promoted the growth of both bacterial strains to some extent or had no discernible effect. Specifically, compared with the blank control treatment, ferulic acid and phenylglyoxylic acid promoted the growth of AS-1 at both cultivation time points, with maximum increases of 13.24% (24 h) and 9.93% (12 h), respectively. After 12 h of cultivation, ferulic acid promoted the growth of AS-1, resulting in a 6.93% increase in growth. However, as the concentrations increased, the effects of these active substances shifted to significant inhibition. At 200 μM, phenylglyoxylic acid and 2-methoxycinnamaldehyde had the most pronounced inhibitory effects on AS-1, with inhibition rates reaching 88.48% (12 h) (Fig. 6a). The effects of the four active compounds on JXF-16 were more complex: compared with the blank control treatment, high concentrations generally caused inhibition, but at 100 and 150 μM (24 h), 2-methoxycinnamaldehyde promoted the growth of this strain (Fig. 6b). In the basic culture medium, low concentrations of active substances had no significant effect on the overall growth of the strains. However, as the concentration increased, all the active substances significantly inhibited the growth of both strains (Fig. 6c).

#### Effects of four active compounds in *C. rotundus* root exudates on AS-1 and JXF-16 biofilm formation

Crystal violet staining was used to measure biofilm formation. For AS-1, results from the 12 h incubation assay demonstrated that phenylglyoxylic acid exhibited a consistent promoting effect across all four tested concentrations compared to the blank control, with increases in biofilm formation ranging from 21.67% to 47.77%. At concentrations of 100 μM and 200 μM, 2-methoxycinnamaldehyde also exhibited a significant promoting effect on biofilm formation by AS-1 (Fig. 6e). When the samples were cultured for 24 h, all four active substances at the four tested concentrations significantly inhibited *R. solanacearum* biofilm formation (Fig. 6e).

For JXF-16, the effects of active substances on biofilm formation were more complex. The results measured at 12 h of cultivation indicated that all four active substances inhibited biofilm formation compared to the blank control (Fig. 6f). After incubation for 24 h, certain substances such as 50 μM p-toluenesulfonic acid, 100 μM ferulic acid, and phenylglyoxylic acid exhibited a significant promoting effect on biofilm formation by JXF-16. However, as the concentration of all the active substances increased, 150 μM phenylglyoxylic acid treatment resulted in an inhibition rate as high as 49.77% (Fig. 6f).

#### Effects of four active compounds in *C. rotundus* root exudates on the motility of AS-1 and JXF-16

**Fig 7:**
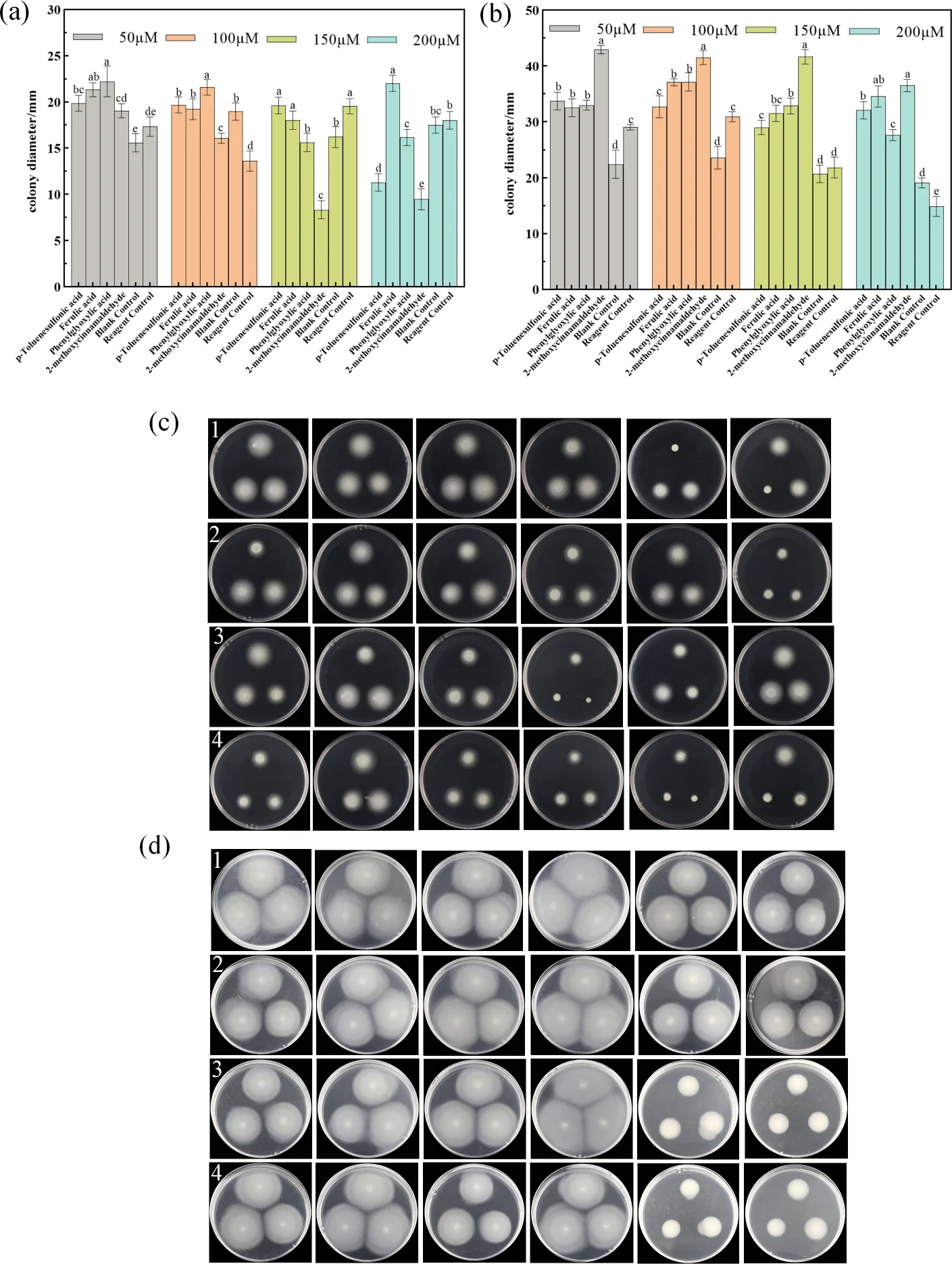
Effects of four active compounds in *C. rotundus* root exudates on the motility of *R. solanacearum* and *B. velezensis.* Effects of root exudates on the motility of (**a**) *R. solanacearum* and (**b**) *B. velezensis*. Panels **c** and **d** show the effects of root exudates on the motility of *R. solanacearum* and *B. velezensis*, respectively. The numbers “1,” “2,” “3,” and “4” labeled in the first column of panels **c** and **d** are only initial layout markers without specific biological meanings, which are set to facilitate the layout distinction between different concentration treatments and control groups. Columns 1 to 4 correspond to treatment concentrations of 50 μM, 100 μM, 150 μM, and 200 μM in sequence. Within each column, the treatments from left to right are listed as follows: p-toluenesulfonic acid, ferulic acid, phenylglyoxylic acid, 2-methoxycinnamaldehyde, sterile water control, and ethanol solvent control.

A semisolid plate culture method was used to determine the effects of various active substances on the motility of strains AS-1 and JXF-16. The effects of the active substances on the motility of the two bacterial strains significantly differed. For *R. solanacearum* (Fig. 7a and c), at a concentration of 50 μM, all the active substances significantly promoted motility compared to the blank control, with the movement diameter increasing by 22.27%–42.68%. However, as the concentration increased, the promoting effect gradually weakened and shifted to inhibition. Among them, 2-methoxycinnamaldehyde showed an inhibition rate of up to 48.58% at 150 μM. For *B. velezensis* (Fig. 7b and d), the four active substances at the four concentrations significantly increased motility. Notably, 2-methoxycinnamaldehyde had the most prominent promoting effect, increasing the movement diameter of *B. velezensis* by 75.91%–101.31%.

#### Validation of the pot control efficacy of *C*. *rotundus* root exudates against bacterial wilt

**Fig 8:**
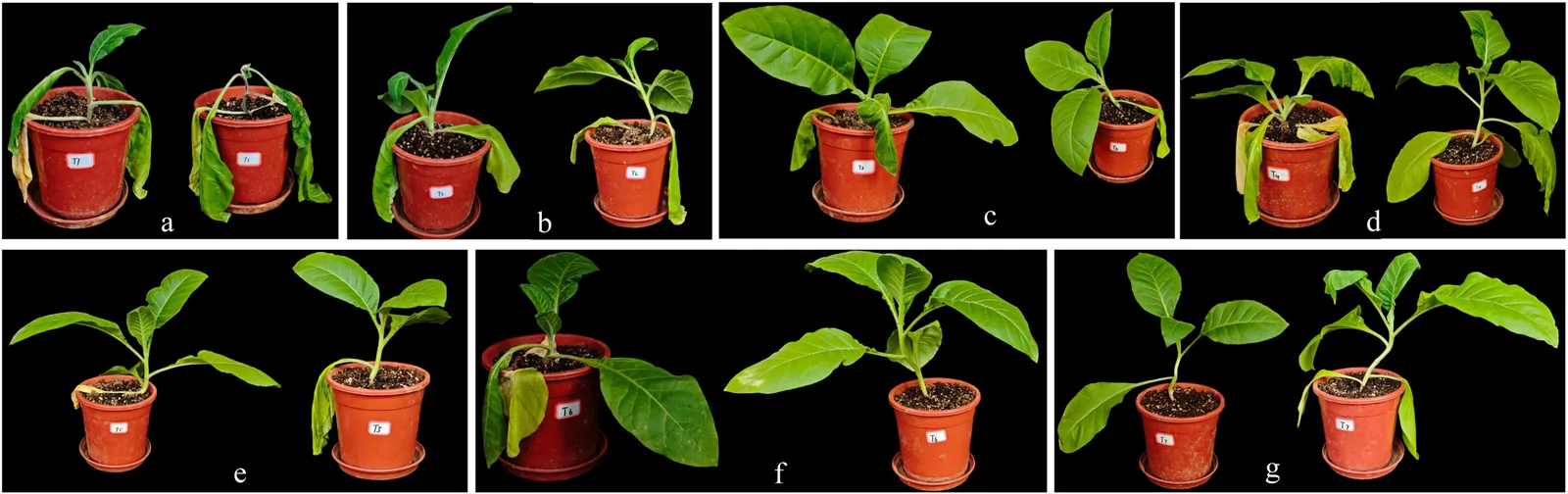
Control effects of different prevention measures on tobacco bacterial wilt. The panels **a**–**g** correspond, in alphabetical order, to the following treatments: CK, 45% kasugamycin–oxine-copper suspension concentrate (SC), JXF-16 bacterial solution, 150 μM phenylglyoxylic acid, 150 μM 2-methoxycinnamaldehyde, 200 μM phenylglyoxylic acid, and 200 μM 2-methoxycinnamaldehyde treatments.

Based on experimental assays evaluating the growth, biofilm formation, and motility of strains AS-1 and JXF-16, we confirmed that 100 μM and 150 μM phenylglyoxylic acid, together with 2-methoxycinnamaldehyde, markedly suppressed the growth of AS-1 but stimulated the proliferation of JXF-16. Additionally, 100–200 μM 2-methoxycinnamaldehyde significantly inhibited AS-1 motility and improved the motility of JXF-16. When applied to potted plants at concentrations greater than 100 μM, phenylglyoxylic acid and 2-methoxycinnamaldehyde achieved a disease control efficacy of over 65% against tobacco bacterial wilt. Accordingly, this study selected 150 μM and 200 μM of phenylglyoxylic acid and 2-methoxycinnamaldehyde for pot-based disease control validation. Experimental results, as shown in Table 2 and Fig. 8, reveal that 200 μM 2-methoxycinnamaldehyde exhibited the most ideal relative control efficacy, with an average relative efficacy of 80.67% across three investigations. Compared to 45% kasugamycin–oxine-copper suspension concentrate (SC), its relative efficacy increased by 16.04 percentage points; meanwhile, compared to the antagonistic bacterial JXF-16 suspension, its relative efficacy improved by 10.49 percentage points. No significant differences in relative efficacy were observed between 150 μM and 200 μM 2-methoxycinnamaldehyde, as well as 200 μM phenylglyoxylic acid. The results indicated that both biocontrol treatment (JXF-16 suspension) and the chemical treatment (45% kasugamycin–oxine-copper suspension concentrate [SC]) had lower relative control efficacies than the active substance treatment groups.

**TABLE 2:** Control results of different control measures on tobacco bacterial wilt^*a*^

Treatment	The average of the three surveys		
	Incidence (%)	Disease index	Relative control effect (%)
CK	58.89	34.94	–^*b*^
45% kasugamycin–oxine-copper suspension concentrate (SC)	36.67	12.72	64.63 ± 3.15d
Bacterial suspension (JXF-16)	33.33	10.37	70.18 ± 1.86cd
Phenylglyoxylic acid (150 μM)	33.33	9.38	72.49 ± 1.91bc
2-Methoxycinnamaldehyde (150 μM)	31.11	8.40	77.71 ± 3.19ab
Phenylglyoxylic acid (200 μM)	33.33	8.15	76.90 ± 2.01abc
2-Methoxycinnamaldehyde (200 μM)	27.78	7.28	80.67 ± 3.21a

^*a*^ Lowercase letters following the values in the relative control effect column indicate significant differences at the 5% level.

^*b*^ “–” indicates not applicable.

## DISCUSSION

High-temperature and high-humidity environments are significant triggers for the proliferation of *R. solanacearum* and the outbreak of diseases. Our findings indicate that as soil temperature and humidity increase, the population of *R. solanacearum* in the rhizosphere of tobacco and *C. rotundus* increases significantly, leading to a higher disease index in tobacco. Concurrently, the α-diversity of rhizosphere bacteria shows a declining trend, a result consistent with the changes in rhizosphere microecology observed in crops such as tobacco (37, 38) and potato (39) following *R. solanacearum* infection. This indicates that pathogen infection disrupts the balance of rhizosphere microbial communities, reducing community stability and disease resistance buffering capacity. Environmental factors further amplify this imbalance, creating a rhizosphere environment that “favors pathogens over beneficial bacteria” (40). Thus, the prevention and control of bacterial wilt should not focus solely on pathogen eradication but should also prioritize the restoration and stabilization of the rhizosphere microecosystem.

The rhizosphere microbial community structure serves as a critical biological barrier against soil-borne diseases. The present study reveals significant differences in the succession trends of rhizosphere communities between tobacco and *C. rotundus* during the disease outbreak period. Overall, the progression of bacterial wilt significantly alters the abundance dynamics of pathogens and antagonistic bacteria. Specifically, the rhizosphere of *C. rotundus* is enriched in beneficial bacteria from the phyla *Acidobacteriota*, *Chloroflexota*, and *Actinomycetota*, resulting in a more stable microbial community structure. Concurrently, *Candidatus Koribacter* and *Granulicella* are significantly enriched in stages A and B of *C. rotundus*, forming important disease-suppressing groups within the *Acidobacteriota*. In contrast, the abundances of *Pseudomonadota* and the *Burkholderia*–*Caballeronia*–*Paraburkholderia* complex, which possess biocontrol potential in the tobacco rhizosphere, decrease significantly as the disease severity increases. Conversely, these groups show an upward trend in the rhizosphere of *C. rotundus*, consistent with the recovery patterns of beneficial rhizobacterial communities observed following the biocontrol of tomato *Fusarium* wilt (41). Additionally, during the disease period, the Chao1 and Shannon indices in the tobacco rhizosphere continue to decline, indicating significant restructuring of the rhizosphere community as the disease progresses (42). In comparison, the rhizosphere community of *C. rotundus* tends to stabilize, suggesting that companion weeds may enhance the disease resistance buffering capacity of the microecosystem by reshaping the rhizosphere community structure (43).

Plant root exudates are key signaling molecules that regulate the construction of rhizosphere microbial communities and the pathogenicity of pathogens. Previous studies have confirmed that citric acid and fumaric acid can promote biofilm formation in Bacillus (44), organic acids and amino acids can recruit beneficial bacteria such as *Pseudomonas* and *Bradyrhizobium* (45), flavonoids can initiate symbiotic signals in rhizobia (46), and continuous cropping autotoxic substances can disrupt microbial balance (47). The composition of exudates varies significantly among plant species (48, 49). As a medicinal plant, *C. rotundus* is rich in secondary metabolites such as alkaloids, phenols, flavonoids, and terpenoids (50), and its root exudates possess the material basis for regulating rhizosphere microorganisms. Previous studies found that extracts from *C. rotundus* rhizomes showed no significant antibacterial activity against *R. solanacearum* (51). In this study, functional validation was conducted on selected active substances from *C. rotundus* root exudates. The results indicated that substances such as phenylglyoxylic acid and 2-methoxycinnamaldehyde exhibit a hormetic pattern—promoting at low concentrations and inhibiting at high concentrations. Low concentrations promote the growth, biofilm formation, and motility of *R. solanacearum*, while high concentrations significantly inhibit these processes. Notably, 200 μM 2-methoxycinnamaldehyde achieved a pot control efficacy of 80.67%, demonstrating potential for development as a novel bio-derived bactericide. In contrast, differential regulation was observed for *B. velezensis*, with some substances promoting its growth at high concentrations. Biofilm formation and motility are core traits underlying the colonization and pathogenicity of *R. solanacearum* (52, 53). Bacterial motility and chemotaxis jointly regulate their environmental adaptability and competitive capabilities, which are critical biological characteristics in microbial ecological interactions (54, 55). These findings suggest that *C. rotundus* may selectively shape the rhizosphere microbiota through its exudates.

In summary, the occurrence and prevalence of tobacco bacterial wilt represent a process of microecological imbalance resulting from the combined effects of environmental conditions, rhizosphere microbial communities, and interactions between the host and companion plants. Specifically, during the pre-disease stage, high temperature and humidity in the soil environment, combined with the effects of root exudates on soil microbial communities, may create conditions for rhizosphere microbial imbalance and disease onset in tobacco. Meanwhile, *C. rotundus* itself relies on exudates to enrich a stable beneficial microbial community, maintaining a healthy state. This mechanism not only explains the phenomenon of *C. rotundus* often coexisting with tobacco in the field without developing the disease itself, but also provides a new perspective for understanding how companion weeds influence crop disease occurrence.

However, this study has certain limitations. The conclusions are only applicable to *in vitro* and pot experiments at the tested concentrations, and the *in situ* content, dynamic changes, and interaction patterns of root exudates from *C. rotundus* and tobacco under natural field conditions have not been clarified. Therefore, follow-up studies should employ *in situ* quantification, non-invasive microsampling techniques, and other methods to accurately measure the actual concentrations and spatiotemporal distribution characteristics of root exudates from both plants and to systematically compare their compositional differences and interaction relationships. Furthermore, integrating multi-omics approaches to analyze the synergistic or antagonistic effects of *C. rotundus* root exudates and their regulatory mechanisms on rhizosphere microbial community structure and function will help clarify the effects of *C. rotundus* on the tobacco rhizosphere microecology, thereby providing a scientific basis for revealing weed-crop interaction mechanisms and the ecological control of bacterial wilt.

## Data Availability

Raw Illumina MiSeq sequencing data were deposited in the NCBI SRA under BioProject accession PRJNA1389825. All publicly available 16S rRNA gene sequences generated in this study have been submitted to GenBank with complete accessible links listed below. The 16S rRNA sequence of R. solanacearum strain AS-1 was deposited in GenBank under accession PX866594. The 16S rRNA sequences of B. velezensis strains JY-5, JY-11, JY-12 and JXF-16 were deposited in GenBank under accessions PX869747, PX869748, PX869749, and PX869746, with valid permanent links. The gyrA gene sequences obtained from Bacillus velezensis strains JY-5, JY-11, JY-12, and JXF-16 have been deposited in GenBank under the accession numbers PZ276144, PZ276145, PZ276146, and PZ276147.
